# Could the significantly increased risk of rheumatoid arthritis reported in Italian male steel workers be explained by occupational exposure to cadmium?

**DOI:** 10.1186/s12995-016-0111-z

**Published:** 2016-05-04

**Authors:** Daniel Murphy, Benjamin James, David Hutchinson

**Affiliations:** Department of Rheumatology, Royal Cornwall Hospital Trust, Truro, Cornwall TR1 3LQ UK

**Keywords:** Rheumatoid, Occupational exposure, Cadmium, Chronic disease

## Abstract

Multiple chronic disease risks have been identified in Italian furnace workers. A range of potential toxins have been identified in foundry dust. We suggest that the heavy metal cadmium (Cd) plays an important role in the development of chronic diseases, notably rheumatoid arthrits, and propose that research into the mechanism of action be undertaken to discover the aetiology of this link.

## Background

We read with interest by Cappelletti et al. “Health status of male steel workers at an electric arc furnace in Trentino, Italy [1]”. The authors demonstrated significantly increased disease risk; specifically, rheumatoid arthritis (RA), hypertension and cardiovascular diseases in exposed workers. The relative risk for RA was observed to be 6.18 (95 % confidence interval (CI) 2.00–19.02, *p* = 0.013). Cappelletti et al. also observed that the foundry dust studied contained iron, aluminum, zinc, manganese, lead, chromium, nickel, cadmium, mercury, arsenic, polycyclic aromatic hydrocarbons, polychlorinated biphenyls and dioxins. Therefore a number of potential toxins may have contributed to the high risk of RA development. We suggest here that cadmium inhalation is a plausible trigger for RA.

## Main text

Cadmium has been demonstrated to be consistently and significantly raised in steel workers [2]. There is an emerging literature to suggest that male RA is associated with a number of occupations associated with cadmium exposure [3]. These include underground mining work (odds ratio (OR) 8.47 (95 % CI 2.59 to 27.66), bricklaying and working with concrete (OR: 2.6, 95 % CI: 1.3–4.9), working with electrics and electronics (OR: 1.8, 95 % CI: 1.0–3), workers exposed to mineral oils (relative risk (RR): 1.4, 95 % CI: 1.0–2.02), workers exposed to hydraulic oils (RR: 1.7, 95 % CI: 1.1–2.6), asphalters (OR: 14.0, 95 % CI 1.2–799.0), and conductors, freight and transport workers (OR: 4.7, 95 % CI 1.4–16.3). Further studies demonstrate a link with smelting and working in metal foundries (OR 2.8, 95 % confidence interval (CI): 1.0–7.4) [4].

Cadmium is inhaled in the work place either as a dust or a fume and is associated with chronic obstructive pulmonary disease (COPD) [5]. It has been suggested that RA is initiated in the lung via the inflammatory process citrullination which involves post-translational changes to proteins and peptides resulting in loss of immune tolerance [6]. Interestingly RA is associates with COPD independent of cigarette smoking. Four retrospective cohort studies with 32,675 RA patients and 122,204 controls were analysed. The pooled risk ratio of incident COPD in patients with RA versus control was 1.99 (95 % CI, 1.61–2.45) [7]. Male RA is strongly associated with smoking which is the most important environmental cause of raised bodily cadmium levels [3]. Moreover, in non-smoking RA patients, hair cadmium levels are consistent with occupational exposure to cadmium (raised 3-fold compared to controls) [8].

## Conclusion

Clear associations between chronic disease and occupations with cadmium inhalation as described by Cappelletti et al. [1] and others [3–5] have been demonstrated. Further epidemiological work related to occupational cadmium exposure and disease development, particularly with regard to RA, is required to modify behaviour influencing development of future chronic disease.

### Ethics approval and consent to participate

Not applicable.

### Consent for publication

Not applicable.

## Abbreviations

CI: confidence interval
COPD: chronic obstructive pulmonary disease
OR: odds ratio
RA: rheumatoid arthritis
RR: relative risk
